# Genomic Study of a *Clostridium difficile* Multidrug Resistant Outbreak-Related Clone Reveals Novel Determinants of Resistance

**DOI:** 10.3389/fmicb.2018.02994

**Published:** 2018-12-06

**Authors:** Joana Isidro, Juliana Menezes, Mónica Serrano, Vítor Borges, Pedro Paixão, Margarida Mimoso, Filomena Martins, Cristina Toscano, Andrea Santos, Adriano O. Henriques, Mónica Oleastro

**Affiliations:** ^1^Departamento de Doenças Infecciosas, Instituto Nacional de Saúde Doutor Ricardo Jorge, Lisbon, Portugal; ^2^Departamento de Genética Humana, Unidade de Tecnologia e Inovação, Instituto Nacional de Saúde Doutor Ricardo Jorge, Lisbon, Portugal; ^3^Instituto de Tecnologia Química e Biológica António Xavier, Oeiras, Portugal; ^4^Centro Hospitalar Lisboa Ocidental, Lisbon, Portugal

**Keywords:** *Clostridium difficile*, multidrug resistant clone, outbreak, resistance determinants, genomic analysis

## Abstract

**Background:** *Clostridium difficile* infection (CDI) is prevalent in healthcare settings. The emergence of hypervirulent and antibiotic resistant strains has led to an increase in CDI incidence and frequent outbreaks. While the main virulence factors are the TcdA and TcdB toxins, antibiotic resistance is thought to play a key role in the infection by and dissemination of *C. difficile*.

**Methods:** A CDI outbreak involving 12 patients was detected in a tertiary care hospital, in Lisbon, which extended from January to July, with a peak in February, in 2016. The *C. difficile* isolates, obtained from anaerobic culture of stool samples, were subjected to antimicrobial susceptibility testing with Etest^®^strips against 11 antibiotics, determination of toxin genes profile, PCR-ribotyping, multilocus variable-number tandem-repeat analysis (MLVA) and whole genome sequencing (WGS).

**Results:** Of the 12 CDI cases detected, 11 isolates from 11 patients were characterized. All isolates were *tcdA*^-^/*tcdB*^+^ and belonged to ribotype 017, and showed high level resistance to clindamycin, erythromycin, gentamicin, imipenem, moxifloxacin, rifampicin and tetracycline. The isolates belonged to four genetically related MLVA types, with six isolates forming a clonal cluster. Three outbreak isolates, each from a different MLVA type, were selected for WGS. Bioinformatics analysis showed the presence of several antibiotic resistance determinants, including the Thr82Ile substitution in *gyrA*, conferring moxifloxacin resistance, the substitutions His502Asn and Arg505Lys in *rpoB* for rifampicin resistance, the *tetM* gene, associated with tetracycline resistance, and two genes encoding putative aminoglycoside-modifying enzymes, *aadE* and *aac(6′)-aph(2″)*. Furthermore, a not previously described 61.3 kb putative mobile element was identified, presenting a mosaic structure and containing the genes *ermG*, *mefA*/*msrD* and *vat*, associated with macrolide, lincosamide and streptogramins resistance. A substitution found in a class B penicillin-binding protein, Cys721Ser, is thought to contribute to imipenem resistance.

**Conclusion:** We describe an epidemic, *tcdA*^-^/*tcdB*^+^, multidrug resistant clone of *C. difficile* from ribotype 017 associated with a hospital outbreak, providing further evidence that the lack of TcdA does not impair the infectious potential of these strains. We identified several determinants of antimicrobial resistance, including new ones located in mobile elements, highlighting the importance of horizontal gene transfer in the pathogenicity and epidemiological success of *C. difficile*.

## Introduction

*Clostridium difficile*, recently renamed as *Clostridioides difficile* (Lawson et al., 2016), infection (CDI), is the main cause of nosocomial antibiotic-associated diarrhea in developed countries, and is prevalent in the healthcare setting. CDI incidence as well as the occurrence of outbreaks has increased dramatically in the last two decades due to the emergence of antibiotic resistant and hypervirulent strains (Freeman et al., 2010; Vindigni and Surawicz, 2015; Isidro et al., 2017). CDI usually develops in hospitalized elderly individuals when the protective colon microbiota is disrupted due to previous antimicrobial therapy (reviewed by Rupnik et al., 2009; Smits et al., 2016). Most *C. difficile* toxigenic strains produce two main virulence factors, the toxins TcdA and TcdB, encoded by genes located in the pathogenicity locus (PaLoc); some strains additionally produce a binary toxin, CDT, while others produce only TcdB (Hunt and Ballard, 2013; Chandrasekaran and Lacy, 2017).

Antibiotic resistance is frequently reported in prevalent *C. difficile* strains and is thought to play a major role in the infection and dissemination of this pathogen, as well as in the emergence of new types of epidemic clones (Spigaglia, 2016; Isidro et al., 2017). Resistance may be due to different mechanisms, such as the expression of genes located on mobile elements or specific mutations in the genes coding for the antibiotics targets (Brouwer et al., 2011; Isidro et al., 2017).

Here we describe a multidrug resistant clone from PCR ribotype 017 *C. difficile* implicated in a CDI outbreak that occurred between January and July 2016 in two surgery wards in a hospital from the Lisbon Metropolitan Area. Multilocus variable-number tandem repeat analysis (MLVA) was used to determine the genetic relatedness of the strains and whole-genome sequencing (WGS) to identify determinants of resistance.

## Materials and Methods

### *C. difficile* Isolates

Following the CDI surveillance program, 11 stool samples from 11 CDI-positive patients, diagnosed using the C. DIFF QUIK CHEK COMPLETE^®^kit, were collected between January and July 2016, during an outbreak in a hospital from the Lisbon Metropolitan Area, and sent to the National Reference Laboratory for Gastrointestinal Infections, hosted in the Portuguese National Institute of Health, for laboratory-based epidemiological surveillance of CDI. As described previously, stool samples were inoculated onto ChromID *C. difficile* agar (bioMérieux, Marcy l’Etoile, France) after ethanol shock and incubated under anaerobic conditions for 48 h at 37°C (Santos et al., 2016). Total DNA was extracted with the Isolate II Genomic DNA kit (Bioline, London, United Kingdom), followed by a multiplex PCR to detect the genes *gluD*, *tcdA*, *tcdB*, *cdtA* and *cdtB* (Paltansing et al., 2007; Persson et al., 2008). An additional PCR was carried out to detect mutations in *tcdA* (Kato et al., 1999). Capillary gel-based electrophoresis PCR ribotyping was performed using Bidet primers, as previously described (Fawley et al., 2015). Patient’s demographic and clinical data was collected by the infection control team of the affected hospital.

### Antimicrobial Susceptibility Testing

Minimum inhibitory concentrations (MICs) of chloramphenicol, clindamycin, erythromycin, gentamicin, imipenem, metronidazole, moxifloxacin, rifampicin, tetracycline, tigecycline and vancomycin were determined with Etest strips (bioMérieux), according to the manufacturer’s instructions. Plates were incubated under anaerobic conditions for 48 h at 37°C. The European Committee on Antimicrobial Susceptibility Testing (EUCAST) breakpoints established for *C. difficile* were used when available. For the remaining antibiotics, the Clinical and Laboratory Standards Institute (CLSI) breakpoints were used (Table 2).

### Multilocus Variable-Number Tandem-Repeat Analysis

Multilocus variable-number tandem-repeat analysis was carried out following the method developed by van den Berg et al. to amplify the loci A6, B7, C6, E7, G8, and CDR60 (Van Den Berg et al., 2007), with an alternative reverse primer to amplify the locus G8, as previously described (Tanner et al., 2010). Each locus size was determined by capillary gel electrophoresis and the corresponding number of repeats was used to construct a minimum spanning tree using the summed absolute distance as coefficient. Isolates with a summed tandem-repeat difference (STRD) ≤ 10 were considered genetically related regardless the number of different loci. Clonal complexes were defined by a STRD ≤ 2 between two isolates that were either single or double locus variants of each other.

### Whole Genome Sequencing and Bioinformatics Analysis

**FIGURE 1:**
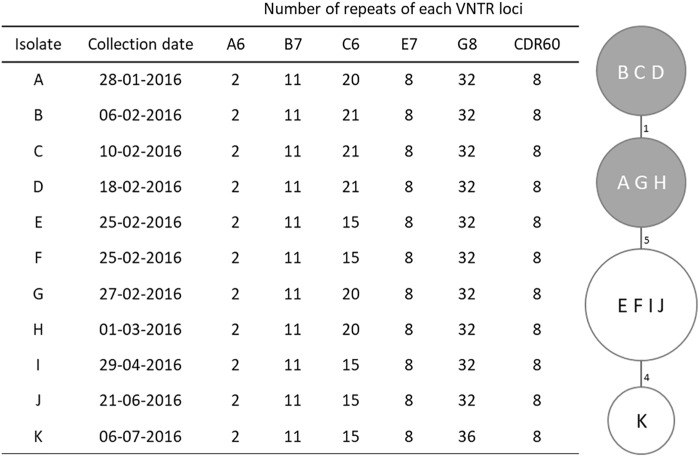
MLVA profiles and minimum spanning tree for *Clostridium difficile* PCR ribotype 017 isolates. For the minimum spanning tree, unique MLVA types are represented by circles, and the summed tandem-repeat differences (STRD) between isolates given by the numbers between the circles. Gray shading indicates a clonal complex (isolates with a STRD of ≤2).

Three strains (A, B, and K; Figure 1) were selected for WGS in order to identify putative determinants of resistance and assess clonal relationship. WGS was performed as previously described (Isidro et al., 2018). Nextera XT libraries were subjected to paired-end sequencing on an Illumina Miseq platform (Illumina Inc., San Diego, CA, United States). After reads’ quality analysis (FastQC v0.11.5^1^) and improvement, (Trimmomatic v0.36), draft genome sequences were *de novo* assembled using SPAdes (version 3.10.1) (Bankevich et al., 2012) followed by annotation using the RAST server^2^ (Aziz et al., 2008). The PubMLST online platform^3^ was used for *in silico* Multilocus Sequence Typing (MLST) and allele determination. Core-genome single nucleotide polymorphism (SNP)-based analysis was performed using Snippy v3.1^4^. Only variant sites with minimum mapping quality of 60, minimum of > 10 reads covering the variant position and > 90% reads differing from the reference genome were considered. Putative antimicrobial resistance (AMR) genes were identified using both CARD^5^ and ResFinder^6^ (Zankari et al., 2012; Jia et al., 2017). Prophage sequences were identified using PHASTER^7^ (Arndt et al., 2016). BLASTn searches^8^ against the non-redundant (nr) and wgs databases were performed to identify the presence (and similarity level) of determinants of resistance in other available genomes. The genome of strain M68 from ribotype 017 (Acc. No. NC_017175) was used as reference. Raw sequence reads of the three *C. difficile* isolates subjected to WGS were deposited in Sequence Read Archive under the Bioproject accession number PRJNA478136.

### Construction of an *ermG* Inducible Strain for Heterologous Expression

To place the *ermG* gene under the control of the anhydro tetracycline-inducible *Ptet* promoter, the *ermG* gene with its ribosome-binding site (positions -12 to + 793 from the translational start codon) was PCR amplified using primers ermG850D (5′ GGATTCGGAGAGGTTATAATGAACAAAG 3′) and ermG1660R (5′ ATAGTTTAGCGGCCGCATTTTAACTTATGCTACCCTACC 3′) and genomic DNA from strain A (Figure 1), isolated in January 2016, from the first outbreak patient, as the template. The resulting 810 bp-long PCR product was cleaved with EcoRI and NotI and inserted between the same sites of pAM25, to yield pMS534. pAM25 is a derivative of pRPF185 from which the *gusA* gene was removed (Fagan and Fairweather, 2011). Plasmids pRPF185 and pMS534 were introduced into *E. coli* HB101 (RP4) and the resulting strains used to transfer the plasmids, by conjugation, into *C. difficile* 630Δerm with selection for thiamphenicol resistance (15 μg/ml) as described before (Serrano et al., 2016). For induction of the *Ptet* promoter, cultures were grown in the presence of 250 μg/ml of anhydro tetracycline (Fagan and Fairweather, 2011).

## Results

### *C. difficile* Isolates

A CDI outbreak occurred between January and July 2016 in two surgery wards of a < 500-bed tertiary care hospital. In 2015, the hospital registered a CDI incidence of 2 cases *per* 10,000 patient bed-days, while there were no cases in the two surgery wards. Twelve cases of nosocomial CDI were detected during this outbreak, 10 in the cardiothoracic surgery ward and two in general surgery ward, with the following temporal distribution: one case in January, seven in February, one in March, one in April, one in June and one in July. The patients’ age ranged from 50 to 84 years and 7/12 were male. According to patient’s hospital medical records, 11 of the 12 patients had received two or more classes of antibiotics in the 3 months prior to the diagnosis. Patient’s demographic and clinical characteristics are summarized in Table 1. The isolates were recovered from 11 of the 12 cases and all belonged to ribotype 017. All were *tcdA*-negative, carrying a previously described ∼1800 bp deletion in *tcdA* (Kato et al., 1999), *tcdB*-positive and did not carry the *cdtA* and *cdtB* genes coding for the binary toxin CDT.

**Table 1:** Characteristics and clinical data of patients with *Clostridium difficile* infection associated with an outbreak.

Patients (*n* = 12) characteristics	Number (%)
%Males	7 (58.3%)
Mean age in years (interquartile range)	71 (64–81)
Ward	
Cardiothoracic surgery	10 (83.3%)
General surgery	2 (16.7%)
Hospital admission during the 6 previous months	4 (20%)
Antimicrobial exposure within 3-months before CDI diagnosis	11 (91.7%)
Classes of antibiotics	
Aminoglycosides	7 (58.3%)
Vancomycin	7 (58.3%)
Carbapenems	3 (25%)
Penicillins associated with clavulanic acid or tazobactam	3 (25%)
Fluoroquinolones	2 (16.7)
Cephalosporins	1 (8.3%)


### Antimicrobial Susceptibility

All isolates showed high level resistance to clindamycin (>256 mg/L), erythromycin (>256 mg/L), gentamicin (>256 mg/L), imipenem (>32 mg/L), moxifloxacin (>32 mg/L), rifampicin (>32 mg/L), and tetracycline (16 mg/L), being susceptible to metronidazole, vancomycin, chloramphenicol and tigecycline (Table 2).

**Table 2:** Antimicrobial susceptibility and determinants of resistance of the 11 *Clostridium difficile* ribotype 017 isolates characterized in this study.

Antibiotic	R breakpoint (mg/L)	MIC (mg/L)	Phenotype (S/R)	Genetic determinant of resistance
Clindamycin	>4**^a^**	>256	R	*ermG*
Erythromycin	≥8**^a^**	>256	R	*ermG*
Chloramphenicol	≥32**^a^**	3–6	S	–
Gentamicin	≥16^**b**^	>256	R	*aac(6′)-aph(2″)* and *aadE^**d**^*
Imipenem	≥16**^a^**	>32	R	Cys721Ser in PBP3**^e^**
Metronidazole	>2**^c^**	0.125–1.5	S	–
Moxifloxacin	>4**^c^**	>32	R	Thr82Ile in GyrA
Rifampicin	>0.004**^c^**	>32	R	His502Asn and Arg505Lys in RpoB
Tetracycline	≥16**^a^**	16	R	*tetM*
Tigecycline	>0.25**^c^**	0.023–0.047	S	–
Vancomycin	>2**^c^**	0.5–0.75	S	–


*^*a*^Breakpoints according to the Clinical and Laboratory Standards Institute (CLSI) interpretative values for anaerobes. ^*b*^Breakpoints according to the Clinical and Laboratory Standards Institute (CLSI) interpretative values for *Staphylococcus spp.*^*c*^Breakpoints defined by the EUCAST guidelines (European Committee on Antimicrobial Susceptibility Testing). ^*d*^Putative mechanism of resistance in other bacterial genera. ^*e*^Putative mechanism of resistance.*

### MLVA

Four MLVA types were identified among the studied isolates (Figure 1), with only one type displaying two loci differences from the remaining. Loci A6, B7, E7, and CDR60 were invariable; C6 was the most variable locus while G8 only differed in the most recent isolate (K). This isolate, from July, displayed the higher distance from the others, with a 10 tandem-repeat difference in loci C6 and G8 from the first isolate, dated from January. All isolates were genetically related and six of them, which had been collected between January 28th and March 1st, constituted a clonal complex (Figure 1).

### Whole-Genome Sequencing Results

The 11 isolates shared a high genetic proximity, as determined by MLVA, and therefore only three, representing the outbreak period and belonging to different MLVA types, isolates A (from January), B (from February, the peak period) and K (from July), were selected for WGS (Figure 1). Data analysis showed the three strains belonged to the multilocus sequence type (MLST) clade 4, ST37. The pathogenicity locus (PaLoc) showed a complete *tcdB* gene (PubMLST allele 9), and a disrupted *tcdA* with a 1.8 kb deletion at the 3′ end and an early stop codon at amino acid 47, which is typical of ribotype 017. Regarding the accessory genes of the PaLoc, no mutations were found in *tcdE*, coding a holin-like protein necessary for toxin secretion, or in the putative negative regulator of toxin production *tcdC* (PubMLST allele 7). The transcriptional regulator *tcdR*, which has a frameshift mutation in the reference strain M68 (locus CDM68_RS03600) due to a deletion at nucleotide 165 that leads to an early stop codon, is in frame, and predicted as functional, in our strains.

Core-genome SNP-based analysis, using the genome of strain M68 as reference, identified a total of 35 single nucleotide variants (SNVs), of which 33 distinguished the strain M68 from the outbreak strains, being that isolates A and B had no differences between each other and isolate K had 2 SNPs distinguishing it from isolates A and B, which is consistent with nosocomial transmission.

WGS data revealed the presence of several determinants of resistance (Table 2). Two genes encoding putative aminoglycoside-modifying enzymes, termed *aadE* (aminoglycoside 6-adenylyltransferase) and *aac(6′)-Ie-aph(2″)-Ia* (bifunctional aminoglycoside N-acetyltransferase AAC(6′)-Ie/aminoglycoside O-phosphotransferase APH(2″)-Ia), were found in the sequenced isolates. BLASTn search against the nr database showed that *aadE* and *aac(6′)-Ie-aph(2″)-Ia*, which are homologous to the loci CDM68_RS08230 and CDM68_RS08245, respectively, in the reference strain M68, are not frequent in *C. difficile* genomes. On the other hand, they are common in other bacterial genera. The gene *aadE* is found with 100% coverage and identity in several *Campylobacter coli* genomes, as well as in a few genomes of *Campylobacter jejuni*, *Streptococcus agalactiae* and *Enterococcus faecalis*, among others. The gene *aac(6′)-Ie-aph(2″)-Ia* found in our isolates is present with 100% coverage and identity in many *Staphylococcus spp*. genomes, but also *Enterococcus spp*. and *Campylobacter spp*, among others.

The tetracycline resistance determinant *tetM* (PubMLST allele 15), homologous to the locus CDM68_RS01945 in strain M68, was also present in our isolates and was identified in the conjugative transposon Tn916 (Acc. No. KC414929).

The substitution Thr82Ile in GyrA (PubMLST allele 35), which is responsible for fluoroquinolones resistance, and two mutations in *rpoB*, leading to the amino acid substitutions His502Asn and Arg505Lys (PubMLST allele 20), both known to be associated with rifampicin resistance, were present in the three sequenced isolates.

Furthermore, we found the mutation 2162G > C in the homolog of locus CDM68_RS05670, which codes for a penicillin-binding protein (PBP), PBP3 (Isidro et al., 2018). This mutation, which leads to the amino acid substitution Cys721Ser, occurs in the PBP transpeptidase domain, the target of carbapenems action (Papp-Wallace et al., 2011).

**FIGURE 2:**
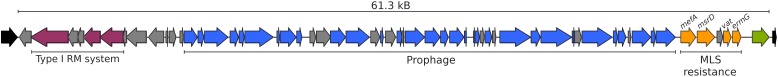
Genetic organization of the novel *Clostridium difficile* putative mobile element harboring the *ermG* gene. Restriction-modification system genes are shown in purple, prophage genes are shown in blue and the genes associated with macrolides, lincosamides and streptogramines resistance are indicated in orange. The transposase is shown in green and the interrupted gene coding for a 23S rRNA (uracil-C(5))-methyltransferase is shown in black. Genes coding for hypothetical proteins are shown in gray.

An *ermG* gene was identified in a cluster of genes associated with macrolide, lincosamide and streptogramins (MLS) resistance that also included the genes *mefA* and *msrD*, both associated with macrolide efflux resistance, and *vat*, coding for a Streptogramin A acetyltransferase (Figure 2). This cluster of MLS resistance genes was found in a 61.3 kb element that interrupts the 23S rRNA (uracil-C(5))-methyltransferase encoding gene (homolog of locus CDM68_RS02190 in strain M68) and shows multiple traits associated with mobile elements likely acquired by horizontal gene transfer (HGT) (Figure 2). This region exhibits a mosaic structure, composed of (i) a Type I restriction-modification (RM) system, with genes coding for the subunits R (restriction), S (specificity) and M (DNA methyltransferase), (ii) an intact prophage of around 49 Kb, as detected by PHASTER, and (iii) the aforementioned cluster of MLS resistance genes, followed by a IS66 family transposase (Figure 2). Three other *C. difficile* genomes deposited in Genbank present this putative mobile element with >99.9% coverage and identity: the non-toxinogenic strain Z31 (ribotype 009) and strains 7499-CF/ST37 and VL_0008, both belonging to ST37 (Acc. Nos. CP013196, MPFV01000002, and CZWM01000001, respectively). Another strain, VL_0387 (Acc. No. FALC01000010), also from ST37, contains a highly similar element (also >99.9% sequence coverage and identity) but in which the region containing the *ermG* and the transposase is inverted, when comparing to the isolates from this study. Seven other *C. difficile* draft genomes (Acc. Nos. FANQ01000006, FAKJ01000001, FADL01000009, FACQ01000001, CZZV01000006, CZYY01000001, CZXE01000001) harbor a similar element (86% coverage and 98.4% sequence identity) that does not contain the MLS resistance portion, which points to the mosaic origin of this element. Likewise, the genome of *C. difficile* strain M120 (ribotype 078) exhibits a ∼40 kb region (Acc. No. NC_017174, genome position 426527–466056) with 62.8% coverage and 90.6% sequence identity with the element present in our strains, while not containing the flanking RM system nor the MLS resistance cluster.

The 61.3 kb putative mobile element has homology with other non-*C. difficile* genomes. For instance, the genomic region spanning the RM system and the prophage has a high homology with two genomes of *Thermoanaerobacter sp*., covering 70% of the element with 88% sequence identity (Acc. Nos. NC_014538 and NC_010320). The proteins coded by the RM system are common in the class *Clostridia* and are also found in *Enterococcus cecorum*. The prophage region is found with 89% sequence identity, covering 62% of the element, in the genome of *Clostridium bornimense* strain M2/40T (Acc. No. HG917868) and the cluster of MLS resistance genes is found in three genomes of *Enterococcus cecorum* with 98.5% sequence identity, covering 9% of the element (Acc. Nos. CP010060, CP010061 and CP010064).

The genes *mefA* and *msrD* present in this element are found with >99% coverage and >95% sequence identity in many bacterial species, most of which are *Streptococcus spp*., mainly *S. pneumoniae* and *S. pyogenes*, but also in *E. cecorum*, *Neisseria gonorrhoeae* and *Acinetobacter junii*, among other species. The *vat* gene is present in a few *C. difficile* genomes and is also found with >96% coverage and >91% sequence identity in several *E. cecorum*, *E. faecium* and *Streptococcus suis* genomes.

The *ermG* gene present in this element is found in multiple species with a sequence coverage and identity ≥99%, including *Lysinibacillus sphaericus* (Acc. Nos. NG_047827 and M15332), *E. cecorum* (mentioned above), *E. faecium* (Acc. No. CP003351), *Bacteroides spp*. (Acc. Nos. NG_047828, L42817, NG_047829.1 and AJ557257) and nine *C. difficile* genomes (Acc. Nos. CP013196, MPFV01000002, FALC01000010, CZWM01000001, FALZ01000014, FAIU01000023, FAES01000003, FACO01000021, FACG01000010), among which is the non-toxinogenic strain *C. difficile* Z31.

**FIGURE 3:**
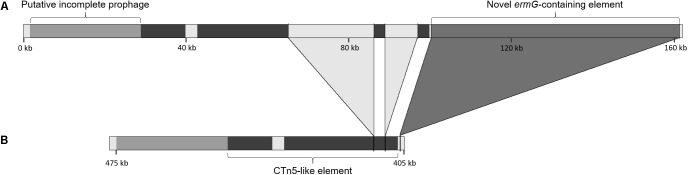
Genetic context of the *ermG*-containing putative mobile element. Genetic context of the novel element containing the *ermG* gene in the strains from the present study **(A)** and comparison of this region with the genome of the reference strain M68 (accession number NC_017175) **(B)**. The darker region represents the homology with the CTn5 element present in strain 630 (accession number AM180355); the light gray regions interrupting this CTn5-like element are not present in strain 630. The two large insertions in the CTn5-like element and the novel *ermG*-containing element differ the strains in this study from strain M68.

The 61.3 kb *ermG*-containing region is absent in reference strain M68 (Figure 3). However, the conjugative transposon Tn6194 harboring the *ermB* gene ( the gene most commonly associated with MLS resistance in *C. difficile*), is present in strain M68, while being absent in all the isolates from this study.

The primer pair ermG-F (5′ TCACATAGAAAAAATAATGAATTGCATAAG 3′) and ermG-R (5′ CGATACAAATTGTTCGAAACTAATATTGT 3′) was used to amplify a 652 bp amplicon of *ermG* and confirmed its presence in the remaining outbreak isolates.

The element containing the *ermG* is located in a region showing evidence of other HGT events (Figure 3), such as prophages and putative conjugative transposons (CTn). Overall, PHASTER identified three complete, one questionable and four incomplete prophages (data not shown). All, except for the complete prophage harboring the *ermG*-element, are found in strain M68. One of the incomplete prophages is located 72 kb downstream the homolog of locus CDM68_RS02190. The 72 kb region between this incomplete prophage and the *ermG*-containing element shows a high homology with the 43.5 kb CTn5 element present in *C. difficile* strain 630 (Acc. No. AM180355, genome position 2137789–2181291). This 72 kb region covers 90% of CTn5 with 99% sequence identity but in the isolates of this study it is interrupted by two genetic insertions of 8 and 22 kb (Figure 3). This 72 kb region is present in the strain BJ08 (Acc. No. CP003939), but in M68 strain it is shorter, lacking the two aforementioned insertions (42 kb; genome position 407967–449991), and more similar to the CTn5 of *C. difficile* strain 630 (Figure 3). The 8 kb insertion shows high homology to a *Campylobacter coli* plasmid (Acc. No. CP017026; 88% coverage, 95% sequence identity), while ∼10 kb of the 22 kb insertion has 99.9% sequence identity with regions of three genomes, namely *Flavonifractor sp.*, *Enterococcus faecium* and *C. difficile* (Acc. Nos. NFHA01000028, LNMU01000054 and MPDX01000112, respectively).

### Confirmation of MLS Resistance Mediated by *ermG*

The *ermG*-inducible *C. difficile* 630Δerm strain was subjected to antimicrobial susceptibility testing by diffusion gradient with Etest strips against erythromycin and clindamycin. Confirming that the expression of *ermG* confers resistance to MLS antibiotics, the MICs of erythromycin and clindamycin were both of >256 mg/L in the *C. difficile* 630Δerm conjugant expressing the *ermG*, when comparing with the MICs observed for *C. difficile* 630Δerm *ermG*^-^ strain (0.75 and 1 mg/L, respectively).

## Discussion

In the present work, we studied a multidrug resistant TcdA-negative *C. difficile* clone from ribotype 017 implicated in a CDI outbreak and identified several determinants of resistance through WGS data analysis. Two novel mechanisms of resistance were described here, namely, the *ermG* gene, which mediates the resistance to MLS antibiotics and is carried by a putative mobile element exhibiting a mosaic structure, and a mutation in a PBP that is likely associated with imipenem resistance.

Ribotype 017 is the most prevalent TcdA-negative *C. difficile* strain and has been considered a recently emerging type, being associated with outbreaks in some European countries (Van Den Berg et al., 2004; Drudy et al., 2007; Goorhuis et al., 2011; Cairns et al., 2015). In a few countries, such as Poland, China or Korea, ribotype 017 is the most common ribotype overall (Pituch et al., 2011; Collins et al., 2013). As such, the lack of one of *C. difficile* main pathogenicity factors (TcdA) does not seem to affect the spreading or infectious potential of these strains.

The described ribotype 017 clone presented resistance to seven classes of antibiotics (Table 2), among which fluoroquinolones, MLS, tetracycline and rifampicin, for which resistance has been described in ribotype 017 in several studies (Barbut et al., 2007; Spigaglia et al., 2011; Dong et al., 2013; Freeman et al., 2015). However, resistance to carbapenems, and its underlying mechanism, is still poorly studied in *C. difficile*. According to a pan-European study, most clinical isolates in Europe are susceptible to imipenem, although ribotype 027 showed elevated MICs compared to other ribotypes (Freeman et al., 2015). Similarly to another clone of ribotype 017 that we described recently (Isidro et al., 2018), the clone characterized in the present study also showed a high-level resistance to imipenem (MIC >32 mg/L).

Resistance to carbapenems in gram-positive bacteria is often associated with single-point mutations in the vicinity of the active site of the PBPs transpeptidase domain, which is carbapenems main target (Davies et al., 2008; Zapun et al., 2008; Papp-Wallace et al., 2011). In this work, we found the mutation Cys721Ser in the transpeptidase domain of PBP3, which is one of the two mutations, along with Ala555Thr in PBP1, that we had previously found in another ribotype 017 imipenem-resistant clone (Isidro et al., 2018). In this previous work, we proposed that these mutations mediate resistance by reducing the binding affinity of imipenem to PBPs. Both the present clone and the one described in the previous study presented a MIC of >32 mg/L but it is possible that their levels of resistance differ at higher concentrations of imipenem, depending on the presence of one or the two mutations, respectively. More studies are therefore needed to fully understand this mechanism of resistance and determine the contribution of each mutation to the resistance phenotype.

Antibiotic pressure can lead to the selection of resistance and promotes the development and spread of resistant strains (Davies and Davies, 2010). Moreover, CDI shows seasonal variation with a higher incidence in winter months, when there is an increase in both hospital occupancy rates and antibiotic consumption due to respiratory infections (Polgreen et al., 2010; Gilca et al., 2012; Brown et al., 2013). Interestingly, in the present study, carbapenems were the most consumed antibiotics in the outbreak ward, with the hospital also reporting a peak of carbapenems consumption during the last trimester of 2015 (data not shown). Altogether, these conditions might have led to the selection and spread of this imipenem-resistant clone, and subsequently to the outbreak, with the first case occurring in January 2016.

Resistance to MLS antibiotics in *C. difficile* is usually due to ribosomal methylation mediated by the rRNA adenine N-6-methyltransferase encoded by *ermB*, and also, but less frequently, by the chloramphenicol-florfenicol resistance gene, *cfr*, which encodes a 23S rRNA methyltransferase that confers resistance to linezolid (Candela et al., 2017). Both these genes are carried by mobile genetic elements such as conjugative transposons (Spigaglia, 2016). The *C. difficile* isolates in the present study were all highly resistant to clindamycin and erythromycin but neither *ermB* nor *cfr* were found by WGS. Instead, the *ermG* gene was found in the genome of all 11 isolates. Additionally, the genes *mefA*, *msrD* and *vat* were also found immediately upstream of *ermG*. The gene *mefA*, firstly identified in *Streptococcus pyogenes*, mediates macrolides resistance by efflux and is common in *Streptococcus spp.* and amongst Gram-positive bacteria in general. The gene *msrD* is associated with the genetic elements carrying *mefA* in *Streptococcus spp*., and can confer the macrolides efflux phenotype in *S. pneumoniae* (Clancy et al., 1996; Daly et al., 2004; Poole, 2005). However, neither *mefA* nor *msrD* confer resistance to lincosamides or streptogramins. Here, we demonstrated that *ermG* expression alone is sufficient to confer a high level of resistance to clindamycin and to erythromycin upon heterologous expression in the ribotype 012 strain 630Δerm.

The *ermG* was located in a novel putative genetic mobile element with a mosaic structure that is not present in the closest reference strain M68 from ribotype 017. This element contained a RM system, a prophage and a cluster of four MLS resistance genes that showed high sequence identity with elements found in other bacterial genus, which is consistent with transmission to *C. difficile* by HGT. This new element is found in very few *C. difficile* available genomes that, however, have no phenotype data available. Although further investigation is warranted, the fact that one of these genomes is from a non-toxigenic strain from ribotype 009 (Pereira et al., 2016) provides strong evidence for the transmission of this *ermG*-containing element between *C. difficile* strains and highlights the importance of non-toxigenic strains as carriers of resistance determinants.

Several studies have showed evidence of interspecies HGT (Bloemendaal et al., 2010; Goren et al., 2010; Juhas, 2015; von Wintersdorff et al., 2016) and *C. difficile* has also been suggested as a reservoir of resistance genes that might be transferred to other species in the human gut (Johanesen et al., 2015). Consistently, our results show a high degree of sequence identity between determinants of resistance found in *C. difficile* and other relevant human pathogens, As an example, in this work we found two genes encoding aminoglycoside-modifying enzymes, *aadE* and *aac(6′)-Ie-aph(2″)-Ia*, that seem to have a low prevalence in *C. difficile* but are widespread in *Enterococcus* spp., *Campylobacter spp.*, *Staphylococcus spp*. or Streptococcus spp. Anaerobes, such as *C. difficile*, however, are naturally resistant to aminoglycosides (which explains the high MICs generally observed) (Khanafer et al., 2018) and hence the presence of these genes may not directly correlate with the resistance phenotype. Nonetheless, the potential transfer of these genes to other species in which they might contribute to aminoglycoside resistance cannot be disregarded. Overall, these results underline the importance of HGT events in the evolution of *C. difficile* and also point to its potential as a resistance reservoir in the human gut (He et al., 2010; Johanesen et al., 2015). This particular multidrug resistant clone of ribotype 017, harboring such a relevant number of determinants of antimicrobial resistance in mobile elements, may likely trigger the dissemination of these determinants in clinical settings as well as in the community and the environment, and thus, it should be targeted by an active laboratory and epidemiological surveillance.

In summary, in this study we described a *C. difficile* multidrug resistant clone implicated in a hospital outbreak presenting new resistant determinants that seemingly promoted the spreading success of this clone. Our data show that *C. difficile* is continually evolving through HGT and indicate that antibiotic selective pressure continues to be a major driving force in the development and emergence of new epidemic strains.

## Author Contributions

All authors contributed to the work described in the paper, as well to the writing and revision of the document.

## Conflict of Interest Statement

The authors declare that the research was conducted in the absence of any commercial or financial relationships that could be construed as a potential conflict of interest.

## Footnotes

1 http://www.bioinformatics.babraham.ac.uk/projects/fastqc/

2 http://rast.nmpdr.org/

3 https://pubmlst.org/

4 https://github.com/tseemann/snippy

5 https://card.mcmaster.ca/

6 https://cge.cbs.dtu.dk/services/ResFinder/

7 http://www.phaster.ca/

8 https://blast.ncbi.nlm.nih.gov/
